# Long-chain Omega-3 Polyunsaturated Fatty Acids in Natural Ecosystems and the Human Diet: Assumptions and Challenges

**DOI:** 10.3390/biom9090485

**Published:** 2019-09-12

**Authors:** Michail I. Gladyshev, Nadezhda N. Sushchik

**Affiliations:** 1 Institute of Biophysics of Siberian Branch of Russian Academy of Sciences, Akademgorodok, 50/50, Krasnoyarsk 660036, Russia; 2 Siberian Federal University, Svobodny av. 79, Krasnoyarsk 660041, Russia

**Keywords:** eicosapentaenoic acid, docosahexaenoic acid, nutritive quality, eutrophication, fish, culinary treatments

## Abstract

Over the past three decades, studies of essential biomolecules, long-chain polyunsaturated fatty acids of the omega-3 family (LC-PUFAs), namely eicosapentaenoic acid (20:5n-3, EPA) and docosahexaenoic acid (22:6n-3, DHA), have made considerable progress, resulting in several important assumptions. However, new data, which continue to appear, challenge these assumptions. Based on the current literature, an attempt is made to reconsider the following assumptions: 1. There are algal classes of high and low nutritive quality. 2. EPA and DHA decrease with increasing eutrophication in aquatic ecosystems. 3. Animals need EPA and DHA. 4. Fish are the main food source of EPA and DHA for humans. 5. Culinary treatment decreases EPA and DHA in products. As demonstrated, some of the above assumptions need to be substantially specified and changed.

## 1. Introduction

**Figure 1:**
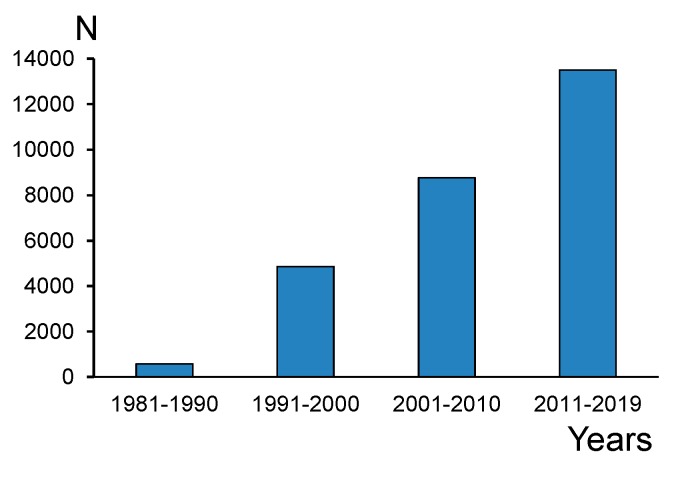
Number of publications containing (*N*) the terms ‘eicosapentaenoic’ or ‘docosahexaenoic’ in the Web of Science Core Collection during the last four decades.

Polyunsaturated fatty acids in the omega-3 family (PUFAs) are a focus of interest in many fields of science: biochemistry, physiology, dietetics, pharmacology, agriculture, aquaculture, ecology, etc. [1,2,3,4,5,6,7]. For many animals and humans, long-chain polyunsaturated fatty acids (LC-PUFAs), namely eicosapentaenoic acid (20:5n-3, EPA) and docosahexaenoic acid (22:6n-3, DHA), are precursors of signaling molecules (bioactive lipid mediators) and essential components of cell membranes in neural and muscle tissues [8,9,10,11]. The number of publications on EPA and DHA in scientific journals has substantially increased since the 1970s (Figure 1). It is impossible to equally review LC-PUFA studies in all the fields of science; therefore, we will primarily take into consideration environmental issues, because natural and agricultural ecosystems are the source of EPA and DHA for human nutrition. There are many papers that report the fatty acid (FA) profiles of diverse microorganisms, plants, and animals, and address EPA and DHA as components of these profiles. However, we will consider only those studies that emphasize the role of these LC-PUFAs as well as their precursors.

In the field of ecology, even when studies have focused on primary producers (microalgae), the importance of EPA and DHA for human health was the rationale for the study of the content and composition of LC-PUFAs [12]. Indeed, only some taxa of microalgae can synthesize large amounts of EPA and DHA, while animals, including humans, have a comparatively low ability for such synthesis via conversion of the precursor, a short-chain PUFA, alpha-linolenic acid (18:3n-3, ALA) [13,14,15]. ALA, which is synthesized by plants, can be obtained by most animals only from food [16,17]. It should be noted that in contrast to EPA and DHA, which are synthesized by some algae, ALA is synthesized by terrestrial vascular plants and is the main component of the photosynthetic membranes of chloroplasts [18,19,20,21]. Since EPA and DHA can be efficiently synthesized *de novo* only by some taxa of algae, aquatic ecosystems are recognized as the main source of these LC-PUFAs in the biosphere [22]. The algae-synthesized EPA and DHA are transferred through trophic chains to organisms at higher trophic levels, invertebrates, and fish, and then to terrestrial consumers, including humans.

In studies of EPA and DHA transfer from microalgae to humans, which inevitably included the culinary treatment of aquatic products for human nutrition, considerable progress has been made in recent decades. Many important findings have been summarized in several keynote statements [1,2,6,12,22], and a number of assumptions have appeared. New data appear continuously, and new questions arise, which naturally challenge some parts of the former assumptions. The aim of this paper is to consider and specify, if necessary, some important assumptions in the field of LC-PUFA production and regarding LC-PUFA transfer from natural ecosystems to the human diet.

## 2. Assumption 1: There Are Algal Classes of High and Low Nutritive Quality

In the nutrition ecology of zooplankton and other microalgaevorous organisms, microalgae have been subdivided into groups representing good and poor nutritive value according to their size and shape, and later to their carbon:nitrogen:phosphorus (C:N:P) ratio [23]. Starting with the milestone work of Ahlgren et al. [24], the PUFA content of microalgae became an important indicator of the nutritive quality of algae for consumers. In this work, it was found that Cryptophyceae and Dinophyceae (Peridinea) had high levels of EPA and DHA, and were the best food for zooplankton [24]. In turn, Chlorophyceae (green algae) and cyanobacteria contained no LC-PUFAs but certain levels of PUFAs, namely ALA, and had comparatively lower nutritive value [24]. Then, in another milestone paper, Muller-Navarra [25] demonstrated that Bacillariophyceae (diatoms), which had a high content of EPA, were a higher-quality food source for *Daphnia* than chlorophytes.

Since then, depending on their content of LC-PUFAs, microalgae have been subdivided into classes representing high and low nutritive quality, Cryptophyceae, Dinophyceae, and Bacillariophyceae vs. Chlorophyceae and cyanobacteria, respectively [26]. Some other classes of microalgae, Eustigmatophyceae, Prymnesiophyceae, Prasinophyceae, Chrysophyceae (golden algae), and Euglenophyceae, have also been shown to have a high content (mg g^−1^ C) of EPA and especially DHA [27,28,29,30]. Based on FA percentages, Trebouxiophyceae and Raphidophyceae were classified as intermediate and excellent food resources for zooplankton, respectively [31]. Recently, the division of phytoplankton classes into four categories was suggested based on the content of several biomolecules that included EPA and DHA: poor, medium, high, and superior quality food [30].

On the one hand, the division of microalgae into classes of high and low nutritive value in terms of their EPA and DHA content is reasonable. Indeed, Chlorophyceae and cyanobacteria (blue-green algae) do not produce these LC-PUFAs [32,33]. On the other hand, species from the classes lacking LC-PUFAs can have a high content of ALA, as mentioned above, and thereby provide a high growth rate for consumers that can efficiently convert ALA to EPA and DHA (e.g., [34,35,36,37] see also Assumption 3 below). For instance, there were no statistically significant differences among the growth rates of *Daphnia magna* when feeding on chlorophytes, chrysophytes, and diatoms [30]. The cited authors emphasized that the reproduction of *D. magna* in feeding experiments was dependent on total n-3 FAs rather than only on EPA.

Moreover, within algal classes that can synthesize EPA and/or DHA, there are species with low contents of these LC-PUFAs and thereby with low nutritive value. Indeed, EPA levels (as a percent of total FA) in 17 marine diatom species used in aquaculture ranged from 5 to 30% [38]. The EPA content in diatoms per gram of organic carbon varied from 1.7 mg g^−1^ C in *Cyclotella meneghiniana* [30] to 45.9 mg g^−1^ C in *Thalassiosira oceanica* [39]. Moreover, in addition to inter-species variability, high variation in EPA content within one species occurs. Indeed, despite the lowest value for *C. meneghiniana* given above, a considerably higher content of EPA, 40.8 mg g^−1^ C, was reported for this species [25]. In natural ecosystems, it has been shown that some marine plankton diatoms have little EPA [40]. In a freshwater reservoir, *Cyclotella* was not associated with the EPA content in seston, while there was a significant correlation between *Stephanodiscus* and the content of EPA [41]. Similar results demonstrating contrasting levels of EPA in different diatom taxa were obtained for river littoral epilithic microalgae [42]. Evidently, diatom species can differ strongly in EPA content, and the common point of view that all diatoms are the superior quality food should be revised.

Consequently, dividing microalgae on the basis of their LC-PUFA content into classes appears to be too coarse for assessing nutritional value for consumers. It is worth noting that ‘nutritional value’ is not characteristic of a food item only, but indicates the demands of the consumer as well. Indeed, if a consumer does not need considerable amounts of EPA and DHA (see also Assumption 3 below), the above subdivision of microalgae into classes of high and low nutritive value is irrelevant. As found in a study of a freshwater plankton community, “there were no phytoplankton species of clearly high or low nutritive value. All phytoplankters, or at least detritus, that originated from them may meet the specific elemental and biochemical requirements of specific groups of zooplankton” [43]. Thus, Assumption 1 should be improved in future studies.

## 3. Assumption 2: EPA and DHA Decrease with the Increasing Eutrophication of Aquatic Ecosystems

The primary producers of EPA and DHA, Bacillariophyceae, Cryptophyceae and Dinophyceae are known to mainly inhabit oligotrophic aquatic ecosystems with low concentrations of total phosphorus (TP), while eutrophic (high TP) aquatic ecosystems are dominated by green algae and cyanobacteria, which do not produce LC-PUFAs (e.g., [44,45]). Thus, a high TP concentration decreases the contents of EPA and DHA in seston due to an increase of cyanobacteria, and thereby decreases the transfer of these LC-PUFAs to higher trophic levels [45,46]. Indeed, the EPA(DHA)-to-carbon content ratio in lake seston had a statistically significant negative relationship with TP concentration in lake water [46], and the EPA + DHA content in perch (per unit muscle mass) also had a statistically significant negative relationship with lake TP [45]. Thus, the nutritive quality of fish for humans becomes lower with eutrophication [45]. An increase in lake eutrophication, measured via the chlorophyll *a* concentration, also resulted in a significant decrease in the EPA content in bighead carp [47].

However, in the above publications, the relative content of LC-PUFAs per unit of organic carbon, C, or fish mass was estimated. These measures of relative content definitely indicate food quality, namely, the nutritive value of seston for zooplankton [46] and fish for humans [45,47]. In addition, if the quantification of the LC-PUFA supply for human nutrition is to be regarded as the paramount aim of relevant ecological studies, it is necessary to quantify this supply as the EPA and DHA yield, Y_LC-PUFA_ value, which has the units μg or mg of LC-PUFA per m^2^ or m^3^ in an aquatic ecosystem per day or year. Thus, to convert food quality, i.e., the relative content of LC-PUFAs (μg mg C^−1^), to the quantity, Y_LC-PUFA_ (μg m^−3^ day^−1^), it is necessary to take into account the production of organic carbon in an aquatic ecosystem, V (mg C m^−3^ day^−1^):

Y_LC-PUFA_ = LC-PUFA ∙ V. (1)

For instance, Muller-Navarra et al. [46] provided the following relation of the content of EPA (μg mg^−1^ C) in lake seston to the concentration of total phosphorus (TP; μg L^−1^) in lake water:

lnEPA = −0.69∙lnTP + 2.78.(2)

From Equation (2), the following dependence can be obtained:

EPA = e^(−0.69∙ln TP + 2.78)^ = 16.12∙TP^−0.69^. (3)

The dependence of the rate of photosynthesis (primary production), V (mg C m^−3^ day^−1^), in lake phytoplankton on TP was given in [48]:

V = 10.4∙TP − 79.(4)

To determine the value of the yield of EPA, Y_EPA_ (μg m^−3^ day^−1^) (Equation (1)), i.e., its amount produced in a lake, it is necessary to multiply Equation (3) by Equation (4):

Y_EPA_ =16.12∙TP^−0.69^ ∙ (10.4 TP − 79) = 167.6∙TP^0.31^ − 1273.5∙TP^−0.69^. (5)

**Figure 2:**
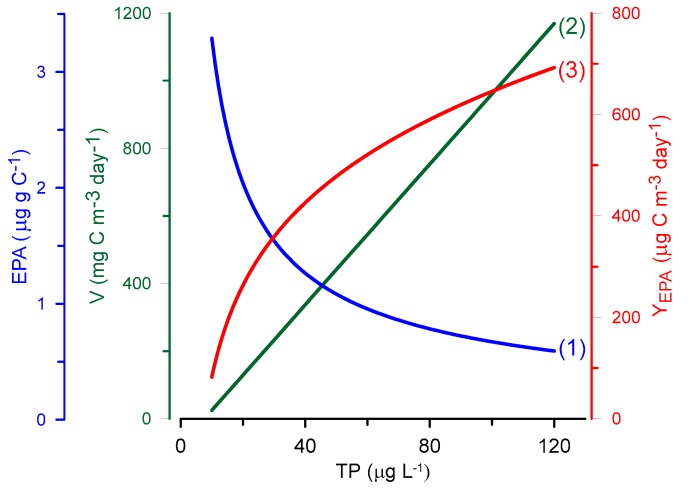
Dependence of the content of eicosapentaenoic acid in lake seston, EPA (1), rate of photosynthesis (primary production), V (2), and the yield of EPA, Y_EPA_ (3), on the total phosphorus concentration in water, TP.

Graphs of Equations (3)–(5) are given in Figure 2. Evidently, with a decrease in the relative content of EPA in seston, there is an increase in EPA yield with increasing TP, i.e., with increasing lake eutrophication. The same is true for DHA if the analogue for Equation (1) is taken from Muller-Navarra et al. [46].

It is worth noting that according to Equation (4), the LC-PUFA yield produced by microalgae (lake phytoplankton) increases gradually with increasing TP (Figure 2). This relationship contradicts taxonomic changes in phytoplankton that usually occur during eutrophication. If primary production in a water body increases only at the expense of cyanobacterial or green algal growth, there would be no increase in LC-PUFA yield. In contrast, the EPA + DHA yield obtained from lakes where perch were caught increased up to a TP concentration of ~40 μg L^−1^ and then decreased with increasing eutrophication [49]. Thus, the maximum LC-PUFA yield (g km^−2^ year^−1^) associated with fish catches occurred in mesotrophic rather than oligotrophic or eutrophic aquatic ecosystems [49].

Moreover, in eutrophic temperate lakes, cyanobacteria dominate in the phytoplankton community only in summer, while in spring, at low water temperatures, “blooms” of psychrophilic diatoms with high contents of EPA often occur. The above equation describing the relation between EPA and TP (Equation (2)) was obtained by Muller-Navarra et al. [46] for summer phytoplankton only. However, if a whole year is taken into consideration, in a eutrophic reservoir, a spring “bloom” of psychrophilic diatoms can produce a large pool of EPA, which can then be transferred through the trophic chain and peak in zooplankton and fish biomass with a time lag during summer [50]. It should also be noted that with decreasing phosphate availability, the proportion of EPA in some freshwater algae significantly decreased [51].

Thus, the common assumption that EPA and DHA decrease with increasing eutrophication of aquatic ecosystems should be improved. Such a decrease occurs in the relative contents of these LC-PUFAs per unit of sestonic organic carbon or fish biomass. However, with regard to EPA and DHA yield, including that available for human nutrition, measured as LC-PUFA quantity per unit area or volume of an aquatic ecosystem per unit time, such a decrease may not occur. At present, mesotrophic aquatic ecosystems are believed to provide a maximum supply of EPA + DHA for humans via fish catches. Ranking aquatic ecosystems on the basis of their ability to produce LC-PUFAs for human nutrition is an important challenge for future research.

## 4. Assumption 3: Animals Need EPA and DHA

As mentioned above, EPA and DHA play important biochemical and physiological roles in humans and many animals, and the common point of view is that all vertebrates and most invertebrate groups require these LC-PUFAs [52]. The low ability of animals to synthesize EPA and DHA from short-chain ALA necessitates them to obtain these LC-PUFAs from food. However, the commonly used terms of “many animals” and “most invertebrates” as well as “low ability” and “necessity” have not been specified or quantified yet. Furthermore, the hypothesis that in natural ecosystems “many” animals can be limited by a low EPA and DHA supply is the important premise of a number of studies [22,52]. Thus, the specification and quantification of the above terms seems to be an important challenge for relevant ecological studies [22,53].

**Figure 3:**
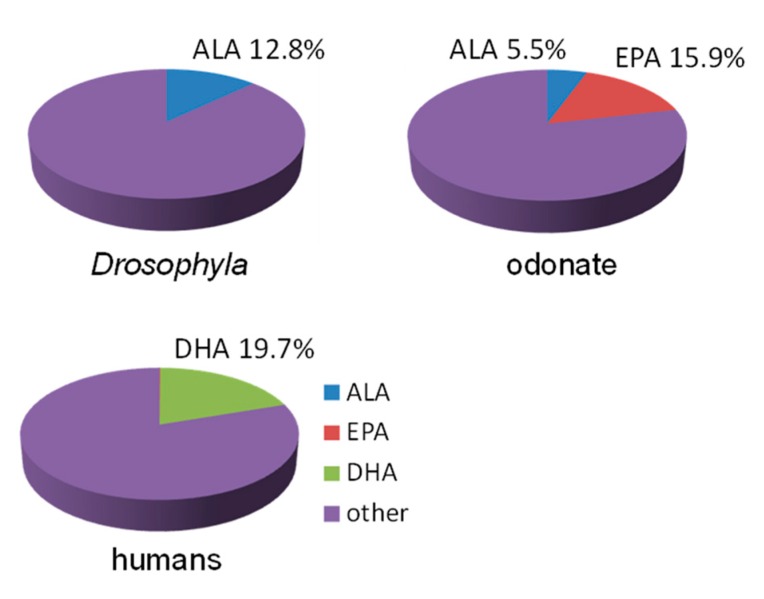
Levels (% of total fatty acids) of alpha-linolenic (ALA), eicosapentaenoic (EPA) and docosahexaenoic (DHA) acids in the heads of *Drosophila* [67], the eyes of odonates [68] and human retinas [3].

First, it should be noted that a large group of invertebrates, terrestrial insects, practically do not have EPA and DHA [54,55,56,57,58,59]. Indeed, terrestrial insects use EPA only as the precursor of lipid mediators, eicosanoids, and thereby synthesize it from consumed ALA in small quantities, at the level of vitamins [60]. In contrast to terrestrial insects, aquatic (amphibiotic) insects have high levels of EPA in their biomass, but contain very low, if any, DHA [61,62,63,64]. As mentioned above, DHA is the main component of the phospholipids of the cell membranes of vertebrate neural tissues, including retinal photoreceptors [3,4,65,66] (Figure 3). However, instead of DHA, there are 18C PUFAs in the eyes of terrestrial insects [3,67] (Figure 3), and EPAs in the eyes of amphibiotic insects [68] (Figure 3). Thus, terrestrial insects evidently do not need EPA or DHA, and aquatic insects do not need DHA in their food or in their biomass in considerable amounts.

Other terrestrial invertebrates, earthworms (*Lumbricus terrestris*), likely need EPA since they have a comparatively high content of this LC-PUFA in their biomass [69]. However, earthworms do not need to obtain these biomolecules from their food, since they likely obtain EPA from their gut microflora [69]. According to our unpublished data obtained using GC-MS and internal standards as in [61,70], Californian worms (*Eisenia foetida*) from a laboratory culture [71] contain 0.37 ± 0.02 and 0.02 ± 0.02 (*n* = 3) mg g^−1^ WW EPA and DHA, respectively. Moreover, some species of soil nematodes can *de novo* synthesize omega-3 PUFAs, namely ALA and EPA [72].

Herbivorous terrestrial vertebrates that consume green parts of plants can satisfy their physiological needs for EPA and DHA through the conversion of ALA [5,15,73]. Some omnivorous terrestrial vertebrates with high metabolic rates, such as the rattlesnake (*Crotalus atrox*), hummingbird (*Archilochus colubris*), white-throated sparrow (*Zonotrichia albicollis*), deer mouse (*Peromyscus maniculatus*), and bank vole (*Myodes glareolus*), have high proportions of DHA in their muscle phospholipids, accounting for up to 33% of the total fatty acids [74,75,76,77]. However, these animals evidently have no dietary source of this LC-PUFA, because even aquatic insects or earthworms, if presented in the diet, could provide them with only EPA rather than DHA.

The high levels of n-3 LC-PUFAs in the functional lipids and organs of some consumers, such as those mentioned above, certainly indicate the physiological significance of these compounds. Recently, new promising approaches have been used to confirm that a consumer has a dietary need for EPA and DHA. One of them combines the elucidation of a dietary source of LC-PUFAs for a consumer and possible physiological consequences of deprivation of this dietary source. For instance, wolf spiders (*Tigrosa georgicola*) that inhabited wetlands and consumed aquatic insects had higher tissue levels of aquatically derived LC-PUFAs and elevated immune function in comparison to upland spiders [78]. Another way to obtain evidence of a dietary need for EPA and DHA is to measure tissue LC-PUFA pools formed due to direct incorporation from an aquatic diet versus conversion from dietary ALA of terrestrial origin. For instance, Twining and colleagues [79] showed that the ALA content of terrestrial insects, and the ALA-to-EPA conversion efficiency, are insufficient to supply insectivorous tree swallow chicks with the n-3 LC-PUFAs that they require. The authors concluded that EPA-rich aquatic insects are ecologically essential resources during a critical ontogenetic period in this bird.

Thus, the statement that “many” animals need LC-PUFAs and must consume them can be challenged by the statement that “many” animals do not need EPA and DHA or do not need to obtain them from food. Evidently, more work is needed to specify species of aquatic and terrestrial animals that truly depend on dietary sources of LC-PUFAs.

## 5. Assumption 4: Fish Are the Main Food Source of EPA and DHA for Humans

Fish and aquatic invertebrates (shellfish, crayfish, etc.) are known to be the main source of LC-PUFAs for humans [7,80,81,82,83,84]. In general, i.e., on a global scale, this statement is absolutely true. However, in some cases, it should be used with caution. First, the contents of the sum of EPA + DHA in the edible parts (muscle tissue) of fish species varied ~400-fold, from 32.78 mg g^−1^ wet weight (WW) in the boganid char (*Salvelinus boganidae*) [85] to 0.08 mg g^−1^ WW in red hybrid tilapia (*Oreochromis* sp.) [86]. To obtain health benefits, the World Health Organization and some national nutrition and health organizations recommend daily personal consumption of 0.5–1.0 g of EPA + DHA [6,7,81,87,88] and even >1.0 g for a Western-type diet [7,88]. Thus, the recommended personal daily dose of EPA + DHA is contained in 15–30 g of meat of boganid char or in 6250–12500 g of meat of the tilapia. Naturally, a question arises: is the fish red hybrid tilapia a real source of LC-PUFAs for humans if 6–12 kg of its meat should be consumed daily to obtain the recommended healthy dose? If 1.0 g of EPA and DHA is considered as the daily dose for the reliable prevention of heart diseases and 1 kg as the maximum portion of fish per serving, the lower threshold value of EPA+DHA content in edible fish biomass is 1.0 mg g^−1^ [85]. All fish species with LC-PUFA contents below this threshold cannot be regarded as a “main source” of EPA and DHA for humans. Nevertheless, these “low LC-PUFA fish” are valuable sources of protein, vitamins, and other nutrients.

Second, some people do not eat fish, and so fish naturally cannot be the “main source” of EPA and DHA for them. For such people, there are alternative dietary LC-PUFA sources, for instance, the livers of terrestrial production animals, which contain EPA + DHA at a level close to the above threshold value of 1.0 mg g^−1^ [83]. However, as mentioned above, on a global scale, the livers of production animals, such as cattle, pigs, and chickens, cannot be alternatives to fish but represent an additional source only. Indeed, the global EPA + DHA supply through the wild fish catch is ~180 10^6^ kg year^−1^, while the global production of both of these LC-PUFAs together in animal livers is ~4 10^6^ kg year^−1^ [83]. In addition, some game birds from the order Passeriformes, which have EPA + DHA contents in their meat above the threshold value, from 1.8 to 3.7 mg g^−1^ [89], may also be an alternative source of these LC-PUFAs for humans who do not eat fish.

Human populations have different diets, e.g., the vegetarian diet in South and Eastern Asia and omnivorous (Western-type) diet in Europe and North America. As a rule, the LC-PUFA status of individuals from populations with a vegetarian diet, i.e., the content of EPA and DHA in various tissues and organs, is significantly lower than that of their omnivorous counterparts [90]. They commonly have a very low intake of preformed LC-PUFAs, ca. 10–74 mg per day, and most EPA and DHA is obtained as a result of the conversion of the dietary short-chain PUFA, ALA [90,91]. The following question arises: how does this conversion meet the demands for LC-PUFAs in vegetarian humans, since its rate is known to be low? Recent studies have found marked global polymorphism in the FADS (fatty acid desaturase) gene cluster, which is strongly associated with the efficiency of the conversion of linoleic and linolenic acids to LC-PUFAs of the corresponding family [92,93,94]. Human populations that have moved to more vegetarian diets are adapted to a low intake of LC-PUFAs, and these alleles provide the more efficient conversion of ALA to EPA and DHA. The selective patterns in FADS genes have been suggested to be driven by a change in the dietary composition of fatty acids following the transition to agriculture. Overall, there is a premise that vegetarian humans can function adequately with the found LC-PUFA status. More studies are necessary to assess the physiological and pathological outcomes of a vegetarian diet in terms of individual- and population-based genetic differences in the metabolism of dietary 18C-PUFAs [91,94].

When discussing fish as the main source of EPA and DHA for humans, the following paradox should be mentioned. Since the growing human population requires an increasing supply of essential LC-PUFAs, and wild catch fisheries are at exploitable limits, a greater proportion of food fish are obtained from aquaculture [95]. However, farmed fish, such as one of the most popular and valuable species, Atlantic salmon (*Salmo salar*), need high levels of EPA and DHA in their diet, which are obtained from the limited wild catch fisheries in fishmeal and fish oil [95]. Thus, in aquaculture oilseed, plant sources are increasingly used in feed to substitute the finite fishmeal and oil. This replacement of fish oil with the sustainable alternative, vegetable oils, has no detrimental effect on fish growth but results in a dramatic decrease in EPA and DHA in their flesh. For instance, the contents of EPA+DHA in the flesh of farmed Atlantic salmon in Scotland decreased from 27.4 mg g^−1^ in 2006 to 13.6 mg g^−1^ in 2015 [95]. Evidently, sustainable sources of EPA and DHA, in addition to fish, must inevitably satisfy the growing human population.

## 6. Assumption 5: Culinary Treatments Decrease EPA and DHA in Products

Polyunsaturated fatty acids are known to be preferentially affected by oxidation during heating [96,97,98]. Thus, the degradation of LC-PUFAs in food during cooking and other culinary treatments has been reported by many authors [99,100,101,102,103,104,105,106,107,108,109,110,111]. However, other authors reported no decrease in EPA and DHA during cooking [70,83,112,113,114,115,116,117,118,119,120,121,122,123]. Indeed, products such as raw fish and production animals do not contain EPA and DHA in pure chemical form, but as components of phospholipids integrated into cell membranes, which have comparatively low susceptibility to degradation [82,124].

It is important to note that the above data on the degradation of LC-PUFAs during culinary treatments are based on measurements of their relative levels as a percentage of total FA. Furthermore, it has been demonstrated that the use of relative values (%) instead of the absolute content of EPA and DHA (mg g^−1^ WW) for the estimation of nutritive value gives erroneous conclusions regarding the nutritive values of fish and other products for humans (e.g., [83,95,117,125,126,127,128]). For instance, there are many fish species with high EPA+DHA contents, >8 mg g^−1^, but a low percentage, <20%, e.g., chum salmon (*Oncorhynchus keta*), coho salmon (*Oncorhynchus kisutch*), and lake trout (*Salvelinus namaycush*), while there are species with a high percentage, >40%, and low content, <3 mg g^−1^, e.g., Atlantic cod (*Gadus morhua*) and whiting (*Merlangius merlangus*) [129].

Another striking example is related to edible macroalgae (seaweeds): red algae (Rhodophyta), (*Palmaria palmata*), have extremely high levels of EPA, ca. 50% of the total fatty acids, but because of their very low total lipid contents, at realistic daily consumption levels, they cannot satisfy the LC-PUFA requirements of humans [126].

There is no correlation, or even a negative correlation, between EPA and DHA levels (%) and contents (mg g^−1^ WW) in fish [129,130]. The explanation for the above phenomenon is believed to be as follows: EPA and DHA are mostly contained in phospholipids (PLs), i.e., in the structural lipids of cell membranes, which should remain nearly constant in proportion to functional muscle tissues, while many other fatty acids are contained in reserve neutral lipids, triacylglycerols (TAGs), whose composition is highly variable in fish biomass [125,130,131,132]. For this reason, in fish which are considered to be fatty, i.e., accumulating comparatively more TAG [133], EPA and DHA are “diluted”, and their relative levels decrease.

Thus, the nutritive value of products for human nutrition should be estimated on the basis of the contents of EPA and DHA, mg per g of product, which can be obtained using internal standards during chromatography, rather than levels, or the % of total FAs [83,117,127,134,135]. Furthermore, content estimates based on internal standards are scarce, and most data are published as the level, or %, of total FAs [100,101,102,105,106,107,108,109,110,111]. Here, we provide data obtained using internal standards, which allows us to compare the real nutritive value of fish and production animal products (Table 1). As noted in Assumption 4, some products prepared from terrestrial animals are comparable in their EPA and DHA contents with those of some fish (Table 1). In general, according to the quantitative data, mg per g of product, there is no decrease in LC-PUFA contents following most culinary treatments, and cooked products prepared from relevant raw biomass are good sources of EPA and DHA for humans.

## 7. Conclusions

A number of assumptions important for developing LC-PUFA studies in the ecological and food sciences should be improved:

1. Dividing microalgae on the basis of their LC-PUFA content into classes of high and low nutritive value appeared to be too coarse. Although there are no Chlorophyceae (green algae) that contain EPA and DHA, there are Bacillariophyceae (diatoms) with low contents of LC-PUFAs.
2. The maximum LC-PUFA yield (g km^−2^ year^−1^) that can be ultimately obtained by humans occurs in mesotrophic rather than oligotrophic aquatic ecosystems.
3. Many animals and terrestrial insects do not need EPA, and aquatic insects do not need DHA in any considerable quantity. Many other animals do not need LC-PUFAs in their food: some worms can obtain these biomolecules from their intestine microflora, and strictly herbivorous terrestrial mammals can synthesize required quantities of EPA and DHA from ALA obtained from the green parts of consumed plants.
4. There are many fish species that are not adequate sources of EPA and DHA for humans, especially for those with a Western-type diet. In turn, there are products of terrestrial animals that can be a source of LC-PUFAs for persons who do not eat fish. In human populations with a vegetarian diet, the conversion of dietary C18-PUFAs is considered to be sufficient to meet the demands for LC-PUFAs based on the found genetic patterns; however, this statement requires further study.
5. Most common culinary treatments do not decrease the EPA and DHA contents in fish and other animal products.

## Figures and Tables

**Table 1:** Contents of the sum of eicosapentaenoic and docosahexaenoic fatty acids (EPA+DHA, mg g^−1^ of product) in cooked fish and the daily portion of products (DP, g) that need to be consumed to obtain the recommended intake of EPA+DHA for humans, 1 g day^−1^.

Product	EPA + DHA	DP	Reference
Atlantic salmon *Salmo salar* (fried)	40.1	25	[121]
Pacific saury *Cololabis saira* (canned, brand H)	37.9	26	[136]
Atlantic salmon *Salmo salar* (fried)	19.6	51	[114]
Pacific herring *Clupea harengus* (canned)	17.9	56	[118]
Atlantic salmon *Salmo salar* (fried)	17.0	59	[112]
Baltic sprat *Sprattus sprattus* (canned)	14.3	70	[118]
Pacific saury *Cololabis saira* (canned, brand N)	13.1	76	[136]
King salmon *Oncorhynchus tshawytscha* (baked)	12.4	81	[137]
Lake trout *Salvelinus namaycush* (baked)	12.4	81	[122]
Lake trout *Salvelinus namaycush* (fried)	12.4	81	[122]
Lake trout *Salvelinus namaycush* (broiled)	12.3	81	[122]
King salmon *Oncorhynchus tshawytscha* (steamed)	11.9	84	[137]
King salmon *Oncorhynchus tshawytscha* (fried)	11.5	87	[137]
King salmon *Oncorhynchus tshawytscha* (microwaved)	10.4	96	[137]
King salmon *Oncorhynchus tshawytscha* (poached)	10.0	100	[137]
Sardine *Sardina pilchardus* (fried)	8.8	114	[112]
Humpback salmon *Oncorhynchus gorbuscha* (boiled)	6.0	167	[116]
Brown trout *Salmo trutta* (boiled)	5.7	175	[117]
Humpback salmon *Oncorhynchus gorbuscha* (stewed)	5.3	189	[116]
Humpback salmon *Oncorhynchus gorbuscha* (roasted)	5.0	200	[116]
Humpback salmon *Oncorhynchus gorbuscha* (fried)	4.3	233	[116]
Brown trout *Salmo trutta* (fried)	4.1	244	[117]
Cod *Gadus morhua* (fried)	4.1	244	[114]
Spanish mackerel *Scomberomorus commerson* (fried)	3.9	256	[112]
Pacific herring *Clupea harengus* (boiled)	3.9	256	[117]
Pacific herring *Clupea harengus* (fried)	3.8	263	[117]
Rock sole *Lepidopsetta bilineata* (boiled)	3.6	278	[117]
Chinook salmon *Oncorhynchus tshawytscha* (fried)	3.2	313	[122]
Rock sole *Lepidopsetta bilineata* (fried)	3.1	323	[117]
Chinook salmon *Oncorhynchus tshawytscha* (baked)	3.1	323	[122]
White sucker *Catostomus commersonii* (baked)	2.3	435	[122]
Cod *Gadus morhua* (boiled)	2.4	417	[117]
Chinook salmon *Oncorhynchus tshawytscha* (fried)	2.8	357	[122]
Cod *Gadus morhua* (fried)	2.2	455	[121]
Walleye (*Sander vitreus*) (baked)	2.1	476	[122]
White sucker *Catostomus commersonii* (broiled)	2.1	476	[122]
White sucker *Catostomus commersonii* (fried)	2.0	500	[122]
Walleye (*Sander vitreus*) (broiled)	1.9	526	[122]
Walleye (*Sander vitreus*) (fried)	1.9	526	[122]
Prawn *Macrobrachium acanthurus* (fried)	1.8	556	[138]
Beef liver (boiled)	1.3	769	[83]
Zander *Sander lucioperca* (boiled)	1.1	909	[70]
Pork liver (boiled)	1.0	1000	[83]
Zander *Sander lucioperca* (stewed)	1.0	1000	[70]
Zander *Sander lucioperca* (fried)	1.0	1000	[70]
Common carp *Cyprinus carpio* (fried)	1.0	1000	[122]
Chicken liver (boiled)	0.7	1429	[83]
Common carp *Cyprinus carpio* (baked)	0.7	1429	[122]
Gilthead sea bream *Sparus aurata* (fried)	0.6	1667	[139]
Common carp *Cyprinus carpio* (broiled)	0.5	2000	[122]
Pork (fried)	0.3	3333	[119]
White rabbit (baked)	0.1	10000	[140]
